# At least two molecules of the RNA helicase Has1 are simultaneously present in pre-ribosomes during ribosome biogenesis

**DOI:** 10.1093/nar/gkz767

**Published:** 2019-09-12

**Authors:** Sivakumar Vadivel Gnanasundram, Isabelle C Kos-Braun, Martin Koš

**Affiliations:** 1 Biochemistry Center, University of Heidelberg, Im Neuenheimer Feld 328, 69120 Heidelberg, Germany; 2 Inserm UMR1131, Institute Universitaire d’Hématologie, Hôpital St. Louis, F-75010 Paris, France

## Abstract

The RNA helicase Has1 is involved in the biogenesis of both small and large ribosomal subunits. How it performs these separate roles is not fully understood. Here we provide evidence that at least two molecules of Has1 are temporarily present at the same time in 90S pre-ribosomes. We identified multiple Has1 binding sites in the 18S, 5.8S and 25S rRNAs. We show that while the Has1 catalytic activity is not required for binding to 5.8S/25S region in pre-rRNA, it is essential for binding to 18S sites. After the cleavage of pre-rRNA at the A2 site, Has1 remains associated not only with pre-60S but, unexpectedly, also with pre-40S ribosomes. The recruitment to 90S/pre-40S and pre-60S ribosomes is mutually independent. Our data provides insight into how Has1 performs its separate functions in the synthesis of both ribosomal subunits.

## INTRODUCTION

Ribosome biogenesis is a complex, highly dynamic cellular metabolic process fundamental to all living cells. In the yeast *Saccharomyces cerevisiae* this pathway begins with the transcription of a large ribosomal RNA (rRNA) precursor, the 35S pre-rRNA in the nucleolus by RNA polymerase I. This large pre-rRNA is further processed into mature 18S, 5.8S and 25S rRNAs through a complex series of endo- and exonucleolytic cleavages and base modifications (methylations and pseudouridylations). The final maturation process takes place in the cytoplasm, where mature ribosomes catalyze the translation of mRNA into proteins. Over 200 non-ribosomal proteins, ∼80 ribosomal proteins and at least 70 small nucleolar RNAs (snoRNAs) are involved in this dynamic process (1–6).

Among the accessory ribosome biogenesis factors are 19 RNA helicases, a large group of enzymes possessing the capability to catalyze the unwinding of double-stranded RNA (dsRNA) by utilizing the energy derived from the binding and hydrolysis of ATP. RNA helicases share a conserved catalytic core, but many have other domains that provide diverse and often still unknown functions. These molecules are found in all kingdoms of life and participate in most steps of RNA metabolism (7,8). Plausible functions of RNA helicases in ribosome biogenesis includes unwinding of snoRNA-pre-rRNA base pairing, remodeling of protein-RNA interactions, pre-rRNA folding and structural rearrangements. The essential *S. cerevisiae* protein Has1 belongs to the DEAD box family of RNA helicases. It is one of factors (such as Rrp5, Prp43 and Spb4) known to participate in the maturation of both ribosomal subunits (9–12). Has1 was implicated in the biogenesis of both 40S and 60S subunits, as its depletion led to the loss of 20S pre-rRNA with accumulation of 35S pre-rRNA and aberrant 23S pre-rRNA, as well as a delay in the processing of 27SB pre-rRNA (9). Additionally, Has1 depletion led to the accumulation of snoRNPs (including U3 and U14, snR10 and snR63 snoRNAs) associated with 90S/60S pre-ribosomal particles suggesting that Has1 is required for the release of some snoRNAs (14). Affinity purifications and proteomic analysis of pre-ribosomal particles indicated that Has1 is associated with the 90S and several pre-60S particles (15–19). Crucially, the ATP dependent unwinding activity of Has1 is known to be essential for its function *in vivo* (13).

To date, only the role of Has1 in the 60S ribosome biogenesis has been extensively studied (20). This report suggests that the presence of Has1 in early pre-60S particle is dependent on the L7, L8 and the set of A3 factors. The authors showed that binding of Has1 to pre-60S particles occurs in an ATP-independent manner and triggers the exonucleolytic trimming of 27S A3 pre-rRNA to generate the 5′ end of 5.8S rRNA. It also suggests that the enzymatic activity of Has1 is needed for the efficient assembly of ribosomal proteins L26, L35 and L37 as well as for the cleavage of 27SB pre-rRNA. However, the role of Has1 in the 90S and biogenesis of 40S ribosomal subunit remains largely unexplored.

Here, we show a detailed analysis of the protein composition of pre-ribosomes purified using Has1 as the bait. We show that Has1 is present not only in 90S and pre-60S ribosomes but also on pre-40S particles. Using RNA-protein crosslinking, we identified multiple binding sites of Has1 on 18S, 5.8S and 25S rRNAs. Our data corroborates the RNA binding sites reported in a new study published during preparation of this manuscript (21). Furthermore, we found that two copies of Has1 are temporarily present in 90S pre-ribosomes and remain associated with both pre-40S and pre-60S after the cleavage of pre-rRNA at the A2 site.

## MATERIALS AND METHODS

### Yeast strains and plasmids

All the yeast strains and constructs used in this study are described in the Supplementary Tables S5 and S6. Unless mentioned all strains were constructed from the parental strain YMK118 (22). Strains for disruption or C-terminal gene tagging were created by PCR based method as described earlier (22). Strains with depletion of essential genes were constructed using TetO7-Ubi-DAA-3xHA cassette (23). All the yeast work and gene manipulations were done following standard methods (24).

### RNA Isolation and northern blotting

The total RNA and northern blotting was performed as previously described (25,26). Briefly, total RNA from an equal number of cells was resolved on a 1% agarose gel and transferred to a Nylon membrane using TV400-EBK Maxi Electro blotter (Severn Biotech). The blot was hybridized with the [^32^P] 5′-end labelled oligonucleotides complementary to pre-rRNAs (listed in Supplementary Table S7) and exposed to phosphorimaging plates which were scanned on a FLA-7000 imager (Fuji).

### Tandem affinity purification

Tandem affinity purifications were performed as previously described (27). Yeast strains were grown at 30°C to an OD600 of 0.8–1.0 in a volume of 2 l of growth medium (YPD/SDC) using a 5 l Erlenmeyer flask with breakers. Cultures were then harvested by centrifugation at 3000g for 5 min at 4°C, washed with pre-chilled milli-Q water and resuspended in lysis buffer (100 mM NaCl, 50 mM Tris–HCl (pH 7.4), 5 mM MgCl_2_, 0.1% TritonX-100, 10% glycerol, 1 mM DTT and protease inhibitor cocktail (Roche, Germany)), then flash frozen in liquid nitrogen. For 100 OD of cells, 200 μl of lysis buffer was used. For lysis, frozen cell pellets were transferred to a ball mill and beaten for 2–3 min using the mixer mill MM 400 (Retsch, Germany). The lysate was then thawed and centrifuged at 20 000g for 30 minutes at 4°C to remove cell debris. The supernatant was incubated with IgG-Sepharose™ (GE healthcare) for 1 h at 4°C on a rotating wheel and then washed with wash buffer (100 mM NaCl, 50 mM Tris–HCl (pH 7.4), 5 mM MgCl2, 0.1% (v/v) NP-40, 10% glycerol, 1 mM DTT). Protein was eluted by TEV cleavage, which was performed by incubating the IgG beads with 1 ml of wash buffer supplemented with 2 μl of AcTEV™ protease (Invitrogen) and incubated at 16°C for 2 h on a rotating wheel. The TEV eluate was subsequently incubated with 50μl of anti-FLAG M2 affinity resins (Sigma-Aldrich) for 1 hour at 4°C and washed thrice. Finally, elution from the beads was carried out by incubating the washed resins with 600 μl of elution buffer (1× flag peptide in washing buffer) for 45 min at 4°C. Purified proteins were TCA precipitated prior to SDS-PAGE.

### Has1 purification of rRNA truncations and quantification

The yeast strain expressing Has1-FTP (YMK688) was transformed with multicopy plasmids carrying different rDNA truncations. Has1 affinity purification was performed as described above and the total RNA associated with Has1 purified complexes immobilized on the IgG-sepharose beads was extracted. The rRNA truncations associated with Has1 were then detected by Northern blot analysis using the ^32^P-5′-end-labeled oligonucleotide complementary to the MS2 tag. SCR1 was used as a purification control. The blots were quantified using AIDA software (Raytek, Germany). The signal of rRNA truncations was normalized to an average of the 18S and 25S rRNAs background level.

### RNA fold-enrichment quantification

To calculate the fold-enrichment of different RNA species in the purification, we averaged the background binding levels of 18S and 25S rRNA. The reason for this decision is illustrated in the Supplementary Figure S6. The use of non-tagged strains to estimate the level of background binding was unsatisfactory, as we obtained very low background, also for 18S and 25S rRNA, significantly lower than what we see in our experiments. The likely explanation is that the affinity tag itself provides a binding surface that can introduce non-specific binding. Therefore, we decided to use several proteins with known function to provide references for specificity and background binding. As can be seen from the figure, Rio2 as a known pre-40S factor did not purify later truncations or truncations after A2 cleavage, while Nsa1 and Lsg1, known pre-60S factors, did not purify the early truncation of 20S rRNA. While some background binding of all different RNA species was observed, in all experiments the highest background binding was that of the 18S and 25S rRNAs. Therefore, the average of their background signals was used to normalize other rRNA intermediates in all experiments. Although this stringent approach may fail to identify *bone fide*, but weak, interactions, we prefer to avoid false positives.

### Mass spec analysis of protein composition by SILAC

For the quantification of protein composition of pre-ribosomes purified through Has1, the stable isotope labeling with amino acids in cell culture (SILAC) technique was employed as previously described [Ong et al. 2002]. Briefly, Has1-WT-FTP strain was grown in a ‘heavy’ synthetic complete media containing ^13^C_6_,^15^N_4_-l-arginine (Arg-10) and ^13^C_6_, ^15^N_2_-l-lysine (Lys-8) (Silantes, Munich). The mutants were grown in ‘light’ media with standard amino acids. For standard SILAC experiments, equal amounts of WT and mutant cultures were mixed and co-purified using the TAP purification approach described above. FLAG elutes were then TCA precipitated, run briefly into a 4–12% gradient SDS-PAGE, trypsin digested and peptide masses were analyzed by nLC–MS/MS (in-house core facility or at Fingerprints Proteomics Facility, University of Dundee, Scotland). The raw data obtained was processed using MaxQuant software (28). The mass spectrometry proteomics data have been deposited to the ProteomeXchange Consortium via the PRIDE (29) partner repository with the dataset identifier PXD013263.

### Sucrose density gradient analysis of affinity purifications

Following affinity purification, 50% of the FLAG eluate was loaded onto a 10–40% sucrose density gradient in lysis buffer (100 mM NaCl, 50 mM Tris–HCl (pH 7.4), 5 mM MgCl2, 0.1% TritonX-100) and centrifuged at 23k rpm for 16 h at 4°C (Beckmann Coulter OptimaTM L-90K ultracentrifuge, SW40 rotor). The remaining 50% was kept on ice, then processed the same way as the fractions and loaded as input on the SDS and northern gels. Following centrifugation, fractions were collected manually and used for protein and RNA analysis.

### Western blotting

Proteins were isolated from the affinity purification and sucrose gradient fractions of proteins by TCA precipitation and then resolved on a 8% SDS-PAGE gel and transferred to Immobilon-FL (Millipore) membrane using wet electro transfer system (Bio-Rad). The membranes were treated with the primary antibodies anti-HA (Abcam), anti-FLAG (gift from Ed Hurt), anti-GST (Santa Cruz), anti-Arc1 (gift from Ed Hurt) followed by anti-rabbit IgG antibody coupled with Alexa Fluor 680 (Molecular Probes) and scanned on the Odyssey Clx imager (Licor).

### UV cross-linking and high throughput analysis of cDNAs (CRAC)

CRAC was performed as previously described (30). Briefly, the culture from the strain expressing Has1-HTP was UV cross-linked *in vivo* using the megatron UV light for 3 min. Following harvesting, the cell pellets were resuspended with lysis buffer (100 mM NaCl, 50 mM Tris–HCl (pH 7.4), 5 mM MgCl2, 0.1% (v/v) NP-40, 10% glycerol, 5 mM β-mercaptoethanol) and lysed by cryo grinding method using the mixer mill MM 400 (Retsch, Germany). The first round of affinity purification was performed by incubating the lysate with IgG-Sepharose™ (GE healthcare, Germany) for 1 h at 4°C on the rotating wheel. Beads were washed with high salt buffers (1000 mM NaCl, 50 mM Tris–HCl (pH 7.4), 5 mM MgCl_2_, 0.1% (v/v) NP-40, 10% glycerol and 5 mM β-mercaptoethanol) and then TEV cleaved for 2 h at 16°C using AcTEV™ protease (Invitrogen, Germany). TEV elute was partially digested with RNace-It™ Ribonuclease Cocktail (Agilent Technologies, Germany) to generate RNAs of a suitable size for library preparation. A second step of purification was performed under denaturing conditions (6M Guanidium hydrochloride) on Ni-NTA agarose (Qiagen, Germany) at 4°C overnight. The purified RNAs were then dephosphorylated using the Calf Intestinal Alkaline Phosphatase (CIAP) (Promega, Germany), and sequentially ligated to L3 linker, 5′-end-labeled and ligated to bar-coded L5 linkers (Integrated DNA technologies), and then eluted using Imidazole (200 mM). Eluates were then resolved on 4–12% gradient SDS-PAGE gel and the band of interest was treated with proteinase-K for 2 h at 55°C, followed by RNA isolation and RT-PCR. The purified amplicon was sequenced on the Illumina platform (Deep sequencing central facility, Bioquant, Heidelberg) and the sequences obtained were aligned to the yeast genome using Geneious software (www.geneious.com) and Py-CRAC suite (30).

## RESULTS

### Has1 is a component of distinct pre-40S and pre-60S particles

**Figure 1.**
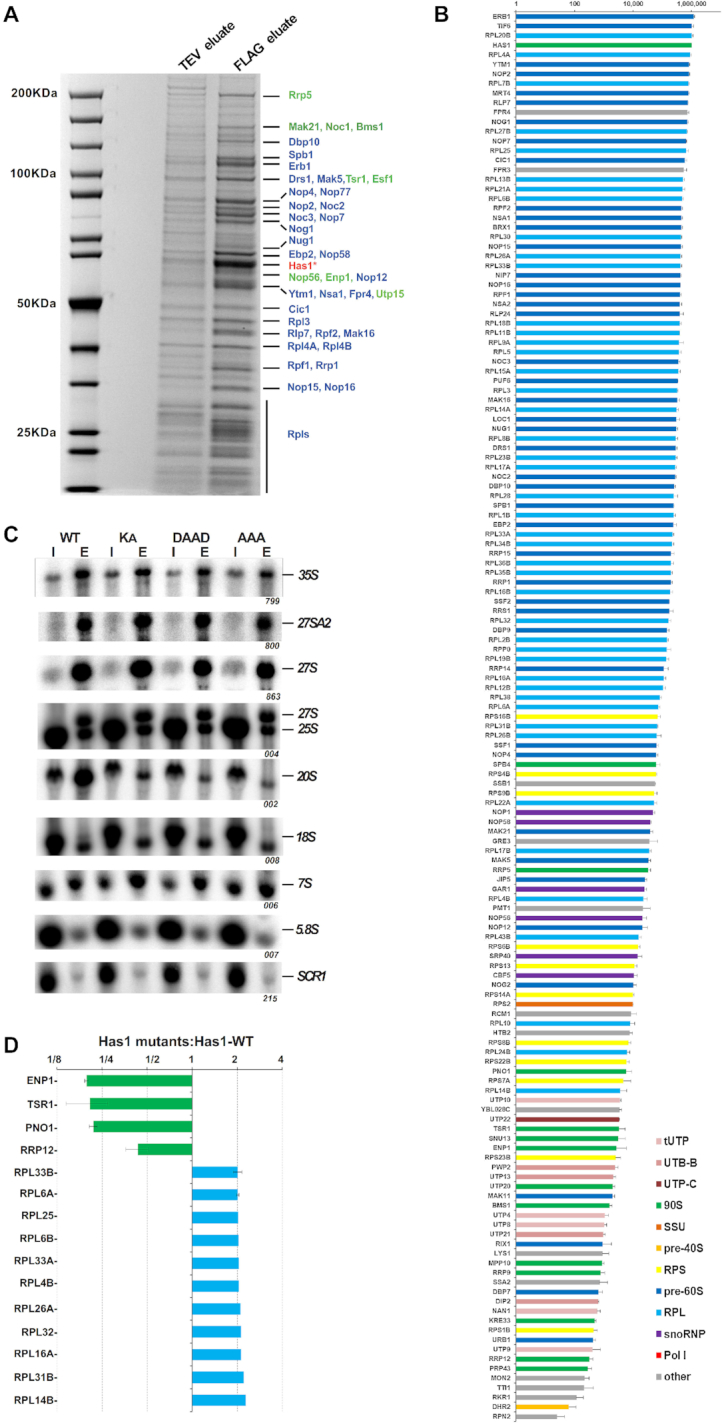
Has1 is a component of both small and large pre-ribosomal particles. (**A**) An SDS PAGE analysis of the proteins co-purified with the wild type Has1-FTP. The TEV (10%) and FLAG eluates were resolved in 4–12% gradient SDS-PAGE and stained by Colloidal Coomassie Blue. The bait protein Has1 is marked with the asterisk. The marked proteins were identified by MALDI-TOF mass spectrometry. Pre-60S factors are depicted in blue and 90S/pre-40S factors in green. (**B**) The protein composition of pre-ribosomes purified via the wild type Has1-FTP. The iBAQ values normalized to bait are plotted (Has1 value was fixed to 10^6^). (**C**) Northern blotting analysis of RNA composition of pre-ribosomes affinity purified via plasmid-borne Has1-FTP (wild type or mutant). The endogenous Has1 wild type was not depleted. (**D**) Proteins with at least 2-fold change in their abundance in the pre-ribosomes purified via the catalytic mutants of Has1. The data from Has1-KA, Has1-DAAD and Has1-AAA experiments were pooled and the average SILAC ratios are shown. Only proteins present in pre-ribosomes from all three mutants were considered. The SILAC H/L ratios were normalized to bait. The error bars represent the standard deviation from the mean H/L ratio of the mutants across experiments.

The RNA helicase, Has1, was implicated in the biogenesis of 40S and 60S subunits and has been identified in purified 90S and early pre-60S pre-ribosomes (9,13,15,18–20). However, the detailed protein composition of pre-ribosomes purified directly via Has1 has not been reported. To determine whether the presence of Has1 in both 90S and pre-60S particles is due to independent roles in the ribosome biogenesis process, we reanalyzed the composition of pre-ribosomal complexes co-purifying with Has1-FTP (FLAG-TEV-protein-A tag), using mass spectrometry and RNA analysis. In total we identified 147 proteins that reproducibly co-purified with Has1-FTP in three independent experiments. Their relative abundance to the bait (iBAQ values) is shown in the Figure 1B and Supplementary Table S1). The complex mixture of ribosome biogenesis factors from different stages indicated that Has1 was likely present in at least three different particles, broadly corresponding to 90S, pre-60S and pre-40S pre-ribosomes (Figure 1A). Has1 was most tightly associated with pre-60S ribosomes, as treating the affinity purified pre-ribosomes with increasingly stringent NaCl concentrations [100–1000 mM] or MgCl_2_ [5–100 mM] led to dissociation of the pre-90S and pre-40S factors but not pre-60S factors (Supplementary Figure S1 and data not shown). Analysis of pre-rRNAs co-purifying with Has1 that showed it to strongly purify the 35S, 27SA/B, 7S and 20S pre-rRNAs, which are components of 90S, pre-60S and pre-40S pre-ribosomes respectively (Figure 1C, left).

It has previously been shown that a catalytically inactive Has1 can be purified with the Rpf2 and Rrp5 containing pre-ribosomes (20). However, the details of when catalytically inactive Has1 enters the ribosome assembly pathwaywas not directly addressed. We therefore analyzed the composition of the pre-ribosomes purified via the catalytically inactive Has1 mutants using SILAC mass spectrometry. We created a yeast strain where the endogenous *HAS1* gene was under the control of repressible TetO7 promoter (TetO7-Ubi-Leu-3HA Has1), allowing fast depletion of the endogenous Has1 upon addition of doxycycline (23). This strain was transformed with plasmids carrying either the wild type or catalytic mutants of Has1 fused to the FTP affinity tag at their C-terminus, under the endogenous *HAS1* promoter. Mutations were made in the conserved RNA helicase motifs I (mutation K92A [KA]), II (E197A [DAAD]) and III (S228A/T230A [AAA]) which were shown to be critical for the ATP binding, ATP hydrolysis and helicase activity respectively (13,31). We hypothesized, that Has1 mutants defective at distinct points of the RNA helicase ATP cycle might arrest the maturation of pre-ribosomes at different stages. The strain expressing the plasmid-borne wild type Has1 was grown in a heavy isotope media and mutants expressing strains in light media. The wild type Has1 was depleted for 4 hours, through the addition of doxycycline. The wild type culture was mixed with each mutant culture in equal ratios and Has1 was purified via the FTP tag, following which the FLAG eluate was analyzed by mass spectroscopy. The overall protein composition of the Has1 pre-ribosomes was very similar between the three mutants, indicating that the three different catalytic mutants did not arrest pre-ribosomes at distinct stages of biogenesis (Supplementary Table S2). Surprisingly, there were also only few significant changes compared to wild type Has1 pre-ribosomes. Figure 1D shows proteins with a minimum 2-fold change in abundance detected in all three mutant pre-ribosomes (as the composition of pre-ribosomes in all three mutants was virtually identical, the data was pooled for simplicity). Four 90S/pre-40S biogenesis factors, Enp1, Tsr1, Pno1 and Rrp12 were clearly reduced, while several ribosomal proteins of the large subunit were enriched in the mutant Has1 pre-ribosomes (Figure 1D).

Next, we analyzed the pre-rRNA composition of pre-ribosomes purified via either wild type or catalytically inactive Has1. As the catalytic activity of Has1 is required for the pre-rRNA cleavage at the A2 site, which is essential for the formation of pre-40S particles, the potential interaction of inactive Has1 with pre-40S pre-ribosomes cannot be analyzed under depletion conditions, since no pre-40S particles are formed. We therefore purified the plasmid-borne wild type or mutant Has1-FTP without depletion of the endogenous wild type Has1 protein and analyzed the pre-RNA composition by northern blotting (Figure 1C right). As expected, wild type Has1 purified the primary transcript 35S pre-rRNA well, in addition to 20S and 27S pre-rRNA intermediates of the 40S and 60S subunits respectively. The catalytic mutants also purified 27S pre-rRNAs equally as well as the wild type protein, however, association with 20S pre-rRNA containing pre-ribosomes was reduced to background levels (Supplementary Figure S2). Intriguingly, the Has1 mutants also purified 27SA2 pre-rRNA, which can be produced only in the presence of fully active wild type Has1. This indicated that either the Has1 is turned over and can be exchanged while in the pre-ribosomes (the mutant Has1 can bind to the 27SA2 containing particle that was produced in the presence of the wild type Has1) or that two molecules of Has1 are present in the early 90S pre-ribosomes. This later scenario appears to be the case, as we describe later.

**Figure 2.**
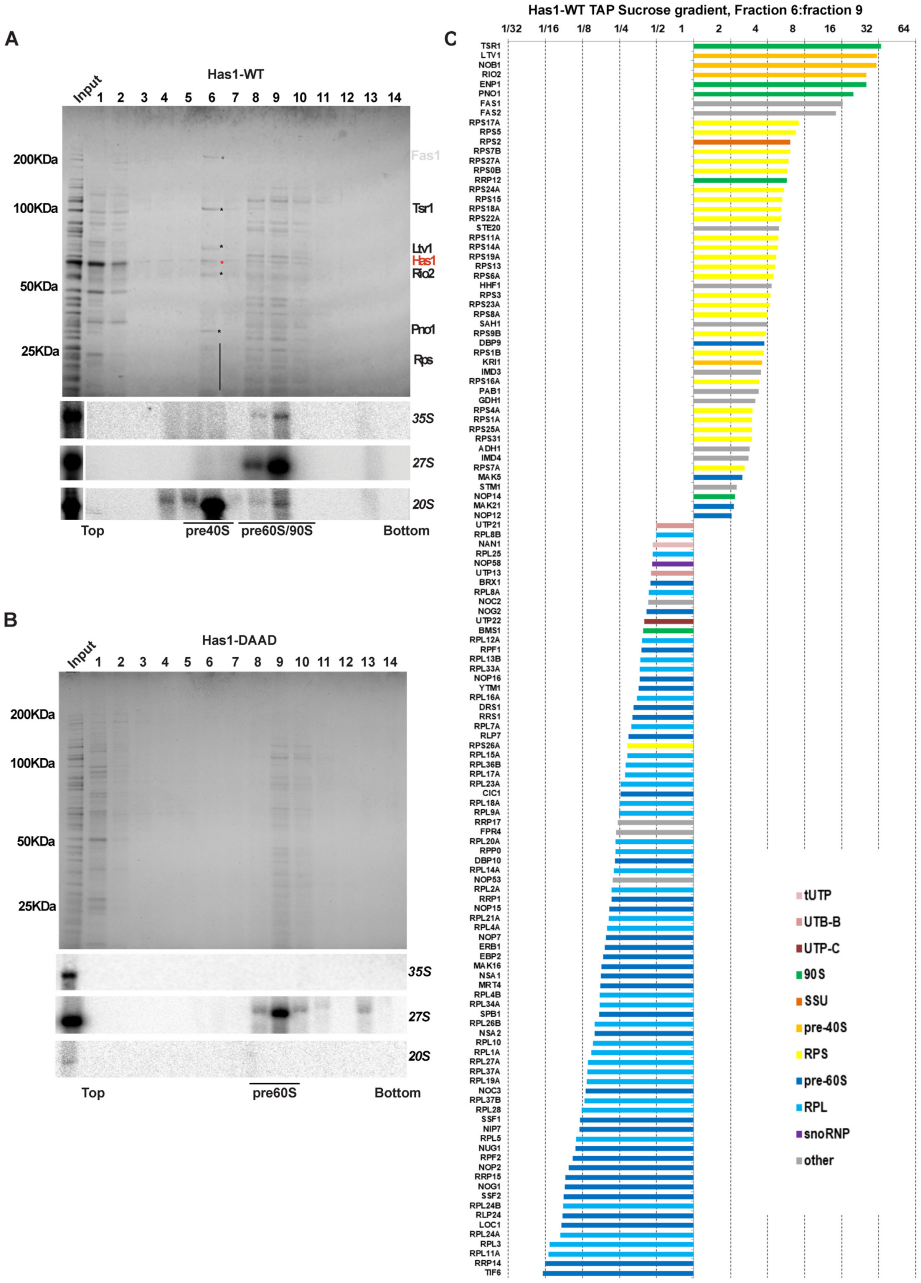
Has1 is present in distinct pre-ribosomes. (**A**) Top: Affinity purified wild type Has1 FLAG eluate was fractionated on the 10–40% sucrose gradient and the proteins were resolved by SDS-PAGE and Coomassie stained. Bottom: northern blotting analysis of RNA in each fraction. (**B**) Same as in A but using Has1-DAAD mutant. (**C**) A ‘spike-in’ SILAC analysis of the protein composition of fractions 6 versus fraction 9 from the purification via wild type Has1. The ratios fraction 6: fraction 9 are shown.

To determine whether Has1 is present in distinct particles the affinity purified wild type or Has1-DAAD mutant pre-ribosomes were fractionated by sucrose gradient centrifugation. Both proteins and RNA were extracted from each fraction and analyzed by electrophoresis (Figure 2A, B). Based on the pre-rRNA species present in each fraction, the wild type Has1 clearly purified the pre-40S (fraction 6) and pre-60S and 90S pre-ribosomes (fraction 8, 9 and 10). Several of the proteins in the pre-40S fraction (6) were present in approximately stoichiometric amounts. These proteins were identified by MALDI-TOF as Tsr1, Ltv1, Has1, Rio2, Pno1 (Figure 2A). In contrast to the wild type, no pre-40S was detectable in the Has1-DAAD purified material (Figure 2B), confirming that catalytically inactive Has1 is not present in pre-40S particles. The lack of 35S signal in the Has1-DAAD gradient fractions is likely due to lower amounts of pre-ribosomes purified by the catalytically inactive Has1, however, the 35S pre-rRNA was clearly detected in the input material (Figure 2B). It is important to note that small amounts of 20S pre-rRNA were also present in the fraction 9 in addition to 35S and 27S pre-rRNA. This presumably represents a transient state of 90S pre-ribosomes immediately after the cleavage at the A2 site when both 20S and 27SA2 pre-rRNAs are still present in one particle.

To analyze the protein composition of different fractions more comprehensively, we used the ‘spiked-in’ SILAC approach. Briefly, the gradient fractions (6 and 9) of Has1-FTP purified from a yeast culture grown in the light isotope media were spiked with equal amounts of the FLAG eluate (total, not separated on a gradient) from a culture grown in a heavy isotope media. This allowed us to directly compare the fractions and identify which proteins are enriched or lost in each fraction compared to total FLAG eluate (input). In agreement with the pre-rRNAs present in each fraction, the lighter fraction 6 consists of predominantly pre-40S factors and small ribosomal proteins whereas pre-60S and 90S factors and large ribosomal proteins are predominantly in the slower sedimenting fraction 9 (Figure 2C and Supplementary Figure S3). We conclude that the wild type Has1 is present in all pre-90S, pre-40S and pre-60S pre-ribosomes, while the catalytically inactive Has1 can associate with pre-90S and pre-60S, but not pre-40S pre-ribosomes.

### Has1 binds to both 18S and 25S regions in the pre-rRNA

**Figure 3.**
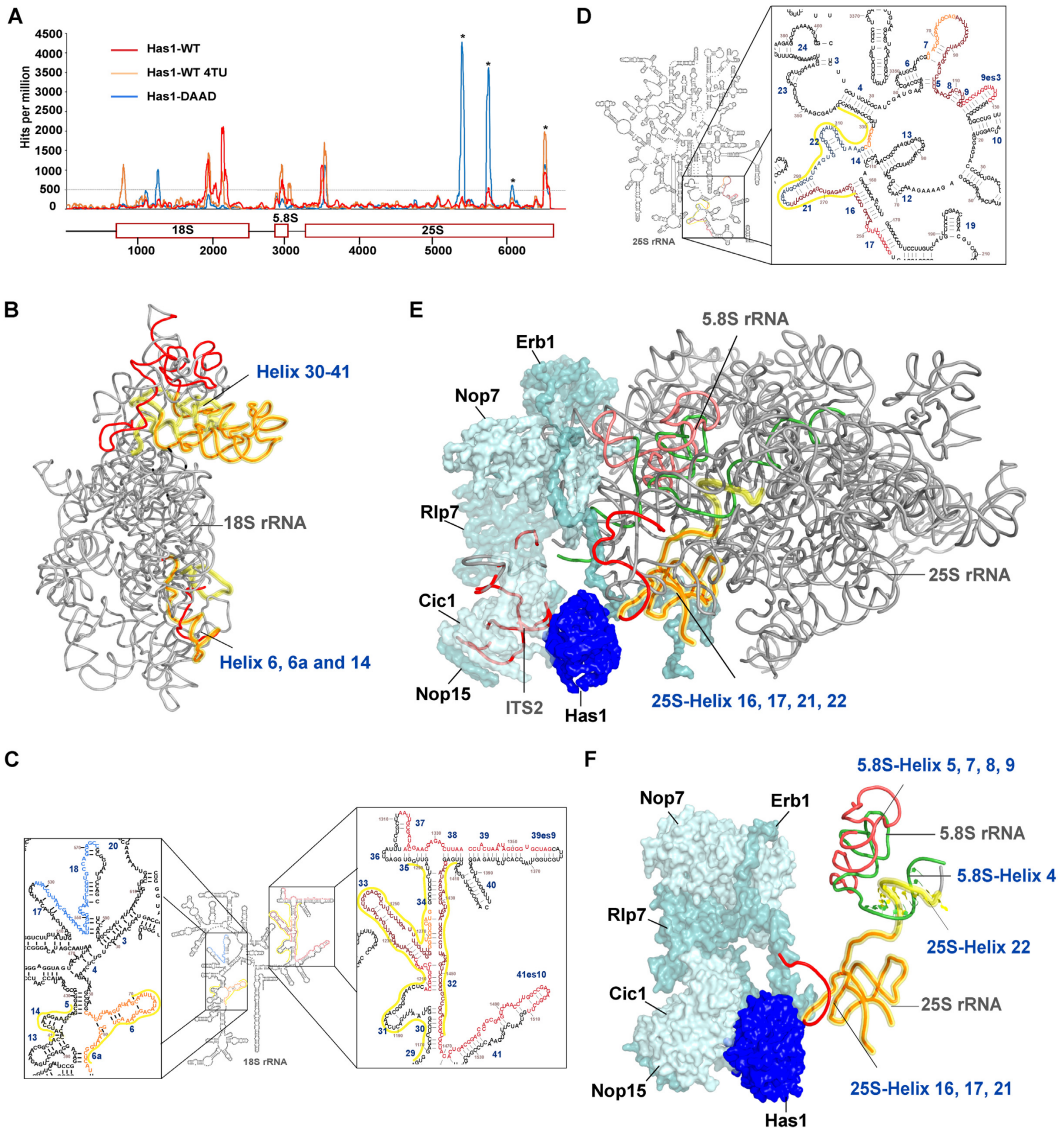
Has1 crosslinks to multiple regions of pre-rRNAs. (**A**) Plot showing the distribution of reads coverage across pre-rRNA. Average number of hits from two independent experiments was plotted. Reads per 1 million total reads are shown. Asterisks indicate common contaminating peaks. (**B**) Binding sites of Has1-WT highlighted in 3D structure of mature 40S (PDB:4V88). (**C**) and (D) Crosslink sites of both Has1-WT and DAAD mutant highlighted in the secondary structure of 18S or 5.8S and 25S rRNAs obtained from RiboVision suite (47): red - Has1 wild type, orange - Has1 wild type 4TU, blue - Has1 DAAD. The overlapping regions of Has1 wild type from standard and 4TU CRAC are shown in dark red. The overlapping regions of Has1 wild type and Has1-DAAD are in dark blue. Has1 binding sites obtained from Brüning *et al.*, (21) are highlighted by a yellow line next to the sequence. (**E**) Crosslink sites of Has1-WT mapped on the 3D structure of pre-60S ribosome (PDB:6EM5). (**F**) Detail of the 5.8S/25S crosslink in the 3D structure of pre-60S ribosome (PDB:6EM1). In the 3D structures, Has1 wild type crosslinked regions are shown in red. The crosslinking regions identified by Brüning *et al.* are highlighted in yellow, with the overlap in orange. The 5.8S rRNA is shown in green and the crosslinked region is in salmon.

As an RNA helicase, it is likely that Has1 contacts RNA directly during its function in ribosome biogenesis. To identify the Has1 binding sites on the pre-rRNA, we performed the UV cross-linking and analysis of cDNA (CRAC) (30) using strains expressing either the wild type Has1 or the catalytically inactive Has1-DAAD mutant. Both proteins were crosslinked to multiple regions in the pre-rRNAs (Figure 3A). The wild type Has1 was crosslinked to the 3′ major (3′ M) domain of 18S rRNA (helices 31–41), the 5.8S rRNA and ITS2 region, and to the 5′end of 25S rRNA (helices 16, 17 and 21, 22) (Figure 3C and D). The Has1-DAAD crosslinking produced overall ∼3 times fewer reads compared to wild type. The crosslinking pattern remained similar to wild type at the 5′ end of 25S rRNA (helices 17–22) but was strongly reduced in the 5.8S and 18S regions. Several new peaks were present in the Has1-DAAD crosslinking profile (Figure 3A). A peak present in the 18S region represents crosslinking to the helices 17 and 18. This region is in the vicinity of U14 snoRNA binding site. Another two regions in the 25S rRNA, corresponding to helices 79 and 89 were strongly amplified, however, these are known from previous studies to constitute contaminating peaks (32,33) and were probably over-amplified due to overall reduced crosslinking efficiency of the mutant Has1-DAAD. In order to obtain better sensitivity of crosslinking, we repeated the crosslinking experiment using 4-thiouridine (4-TU) in the culture media. The wild type Has1 produced very strong crosslink (about 9x the number of reads) with the profile very similar to the results obtained from the standard CRAC experiment (Figure 3A). Interestingly, a new crosslinking site was detected in the 18S rRNA region, helices 4 and 6. Unfortunately, we were not able to crosslink the mutant Has1-DAAD using 4-TU for reasons that remain unclear. The binding sites of Has1 in 18S rRNA are in close proximity to the known binding sites of other pre-40S processing factors (Enp1, Ltv1, Nob1 and Rio2), while the binding sites of Has1 in the 25S rRNA are in the vicinity of binding sites of Erb1, Nop7, Nop12 and Nop15 (32,33) (Supplementary Tables S3 and S4). To better understand the spatial arrangement of the Has1 crosslinking sites, we highlighted the observed sites of crosslink in the available 3D structures of either the mature 40S or pre-60S ribosomes (Figure 3B, E and F). Unfortunately, the currently available 90S or pre-40S ribosome cryo-EM structures lack sufficient resolution to see the all the Has1 crosslinking sites. To allow a direct comparison with our results, the recently reported Has1 crosslinking sites identified using PAR-CRAC by Brüning *et al.* (21) are highlighted in yellow in both 2D and 3D structures, with overlapping crosslinking regions depicted in orange in the 3D structures. As can be seen the crosslinking pattern is in very good agreement with the results of Brüning *et al.* (21). In addition to the sites in pre-60S identified by Brüning *et al.*, we also see a clear crosslinking to ITS2, which is in immediate vicinity of Has1 in the cryo-EM structure (6EM5) (34) Figure 3E. Interestingly, Brüning *et al.* observed only low levels of crosslinking to 5.8S rRNA region, which clearly crosslinked to Has1 under our conditions. However, they observed an extended crosslinking site within the 25S rRNA helix 22, across the portion which forms a helix with 5.8S rRNA, as shown in the Figure 3F. Thus, the crosslinking sites in 5.8S rRNA observed by us and the 25S rRNA site observed by Brüning *et al.* are adjacent in pre-60S ribosomes and likely represent the same Has1 binding site. We conclude that our results corroborate the results of Brüning *et al.* and *vice versa*.

Taken together, these results support the observation that Has1 can participate in the rRNA processing by directly interacting with rRNA of both small and large ribosomal subunits. The distance between the different crosslinking sites in the 18S rRNA are rather far apart in the 3D structures. It is therefore plausible that Has1 is recruited to these sites separately (see the discussion for further details).

### Two Has1 molecules temporarily coexist in 90S complex

**Figure 4.**
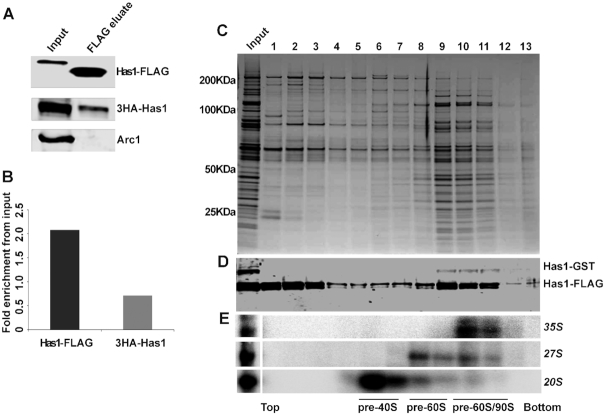
Has1 is present in multiple copies in 90S pre-ribosomes. (**A**) Western blot analysis of Has1-FTP purification from the yeast strain expressing both 3HA-Has1 and Has1-FTP using anti-HA, anti-FLAG and anti-Arc1 antibodies respectively as indicated. The change in the molecular size of Has1-FLAG is due to the TEV cleavage during tandem affinity purification. (**B**) Chart showing the average fold enrichment of 3HA-Has1 and Has1-FLAG based on the quantification of data from two independent experiments. (**C**) Sucrose density gradient analysis of the FLAG eluate from affinity purification using the strain expressing both Has1-GST and Has1-FTP. Proteins isolated from each fraction were resolved by SDS PAGE and detected by Coomassie staining. (**D**) Western analysis of the fractions using the anti-Has1 and anti-GST antibodies. (**E**) Northern analysis of RNA in each fraction. The fractions corresponding to different pre-ribosomes based on the pre-rRNA content are indicated below the image.

Since Has1 co-purified early 90S, pre-40S and pre-60S factors and also interacted with multiple sites in 18S and 5.8S/25S rRNA, it is plausible that Has1 is present as two independent copies in pre-ribosomes. This would also explain the observed purification of 27SA2 by Has1-mutants (see above and Figure 1C). To investigate this hypothesis, the yeast Has1 depletion strain YMK444 (expressing 3HA-Has1 under the control of TetO7 promoter) was transformed with a plasmid carrying Has1-FTP under the control of the wild type Has1 promoter. In principle if two copies of Has1 are present in the same pre-ribosomal particle, then the affinity purification through one copy of Has1 should co-purify the other. We purified pre-ribosomes via the Has1-FTP and analyzed them for presence of the 3HA-Has1 by Western blotting. The tRNA binding protein Arc1 was used as the negative control. As can be seen in the Figure 4A, the 3HA-Has1 clearly co-purified with the Has1-FTP, confirming that at least two copies of Has1 RNA helicase are simultaneously present in the same pre-ribosomes (Figure 4A and B). The same result was also obtained in a strain co-expressing a Has1-GFP and Has1-FTP (Supplementary Figure S4).

To determine, which pre-ribosomes contain multiple copies of Has1 we fractionated pre-ribosomes purified via the Has1-FTP by size using the sucrose-density gradient centrifugation (Figure 4C, D). To be able to better distinguish different Has1 copies in the Western analysis, we used a yeast strain with the genomic copy of Has1 tagged with GST at the C-terminus and expressing the plasmid-borne Has1-FTP. Corroborating the previous observation, Has1-GST was co-purified with the Has1-FTP (Figure 4D). Importantly, the Has1-GST was detected only in the fractions 10–11 corresponding to the 90S pre-ribosomes (based on the presence of 35S pre-rRNA) and in the fraction 9 corresponding to the pre-60S particles containing 27S pre-rRNA (Figure 4E). Notably, the fractions 9–10 contained in addition to 27S pre-rRNA also small amounts of 20S pre-rRNA (Figure 4E top) suggesting that at least two copies of Has1 remained temporarily in the same pre-ribosomes after the A2 cleavage.

### Recruitment of Has1 to pre-40S and pre-60S/90S is mutually independent

**Figure 5.**
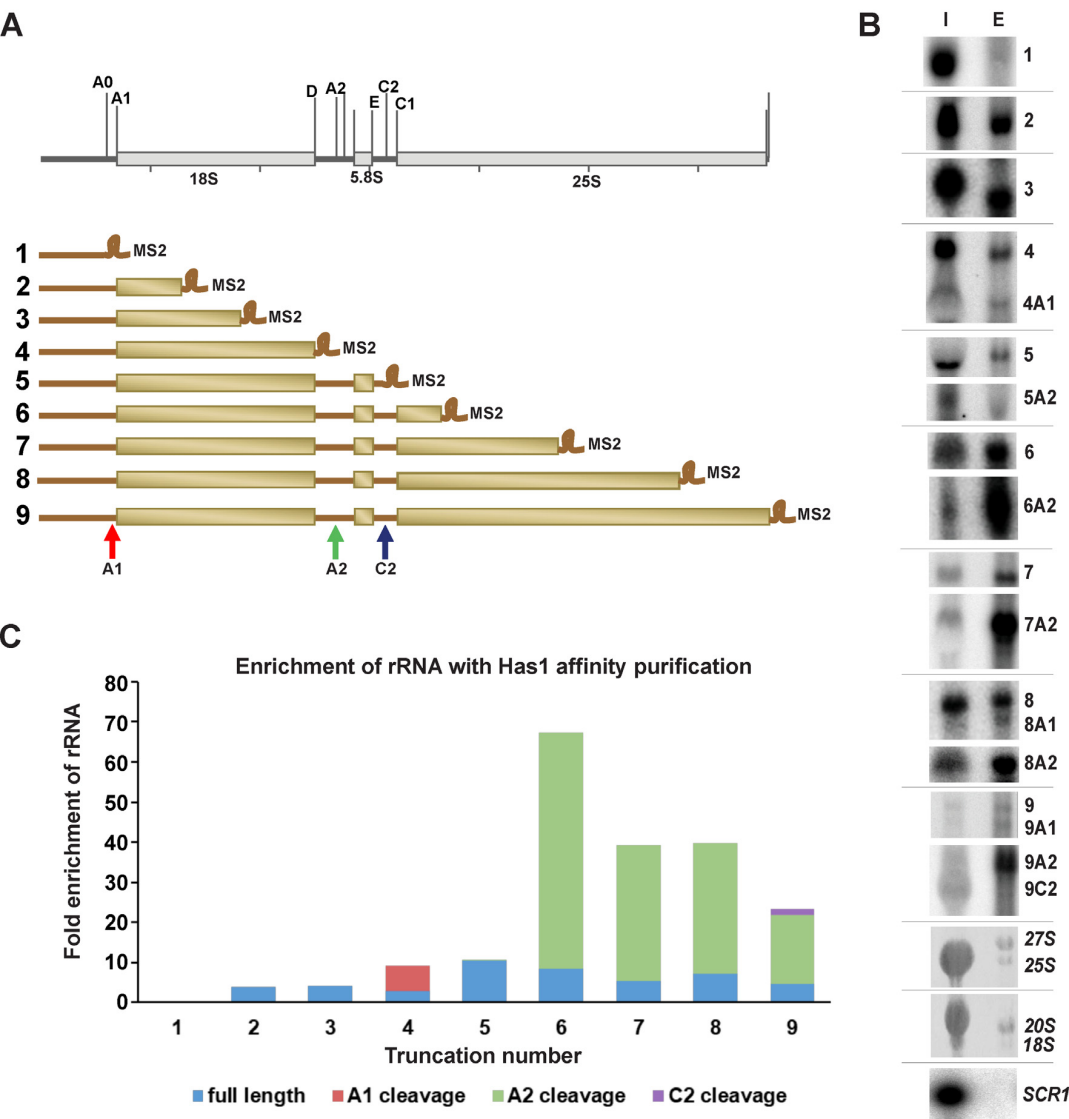
Timing of Has1 recruitment to preribosomes. (**A**) Schematic representation of the 35S rDNA and various rDNA truncations with MS2 tag used. (**B**) Northern blot analysis of rRNAs co-purifying with Has1-FTP using a probe against the MS2 tag. Lane I – 1% of the input, lane E – total RNA purified with Has1. (**C**) Quantification of the fold enrichment of pre-rRNAs purified through Has1-FTP. The full length rRNA and the cleaved ones are indicated with different color codes. The uncropped northern blot is shown in the Supplementary Figure S5.

To determine whether the recruitment of Has1 into pre-ribosomes occurs at one or multiple time points, we employed a set of rDNA truncations plasmids. Briefly, yeast strains expressing Has1-FTP were transformed with a set of multicopy plasmids that carry a copy of the whole rDNA unit truncated at different distances from the transcription start site through the introduction of a hybridization tag (corresponding to a MS2 binding site) (Figure 5A). These truncations are expressed from the native RNA polymerase I promoter and the transcription terminates at the native terminator, ensuring that processing of the pre-rRNAs synthesized from these rDNA truncations is as physiological as possible. The rRNA truncations that co-purified with Has1-FTP were detected by northern blotting analysis using a probe complementary to the hybridization tag (Figure 5A, B and Supplementary Figure S5). The SCR1 (small cytoplasmic RNA 1, an abundant cytoplasmic RNA, the RNA component of the signal recognition particle) RNA, was used as the loading control. Has1 did not associate with the rRNA truncation which contains only the 5′ ETS and showed clear enrichment only for the truncations containing the 5′ETS and 18S rRNA/ITS1 regions. However, Has1 very strongly co-purified the rRNA truncations containing the 5′ end of the 25S rRNA (1–421nt) (Figure 5C). These results suggest that Has1 is recruited to pre-ribosomes after the complete 18S rRNA is transcribed, and mainly upon transcription of the 5′end of the 25S rRNA.

**Figure 6.**
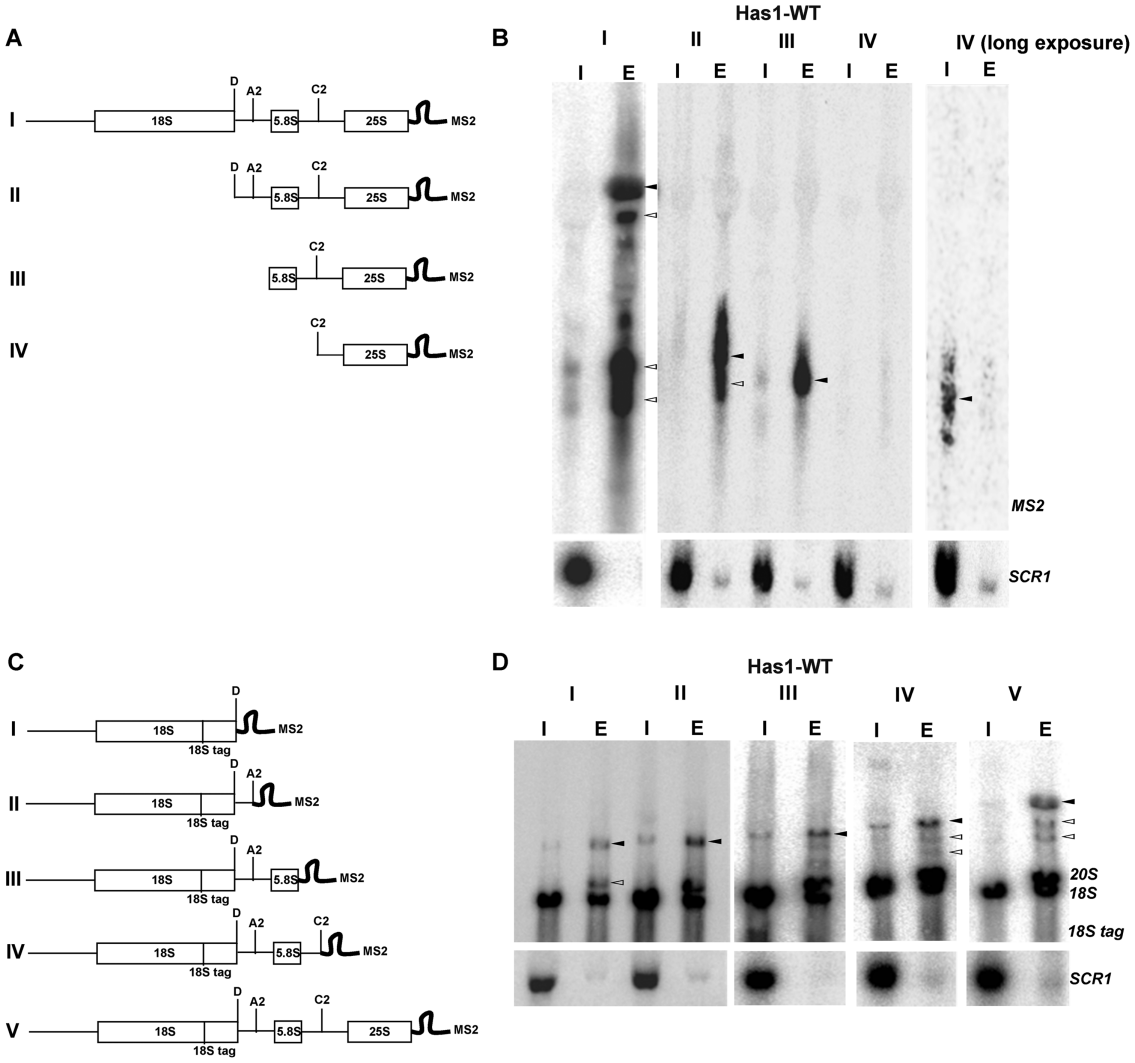
Has1 can be recruited to 18S and 25S regions in pre-rRNA independently. (**A, C**) Schematic representation of the rDNA truncations used. (**B, D**) RNA co-purified with Has1-FTP was analyzed by northern blotting using probes against the MS2 tag or 18S-tag respectively. Probing for SCR1 RNA was used as the purification control. Lanes I - 1% of the input, lanes E - total eluted RNA purified with Has1-FTP. The black arrows indicate the full-length transcripts of each truncation. The white arrows indicate transcripts partially processed by cleavage at sites A0/A1, A2 and C2 respectively.

To examine if the recruitment of Has1 to the pre-60S and pre-40S is independent, we made truncations containing only the 5′end (1–421nt) of 25S rRNA together with or without the 18S rRNA, ITS1 or 5.8S regions (Figure 6A and C). To allow us to detect the 5′ part of the pre-rRNA after the A2 cleavage, an internal oligonucleotide tag was inserted into the 18S rRNA sequence as previously described (35). Has1 was recruited to the 25S rRNA truncations lacking the 18S rRNA and/or ITS1 regions, however it failed to associate with the truncation lacking 5.8S rRNA and intact ITS2 regions (Figure 6A and B). Therefore, the recruitment of Has1 to pre-60S particles does not require presence of the binding sites in the 18S rRNA. This is in agreement with previously published data obtained by Chen et al 2017 (36). Similarly, for the binding to the 18S portion of pre-rRNA, as can be seen from the Figure 6D, Has1 can bind truncations containing solely the 18S rRNA, however, the interaction is enhanced when the ITS2 and 5′end of 25S rRNA are included (construct V), in agreement with the results in the Figure 5C. Taken together the data presented here allow us to conclude that at least two distinct molecules of Has1 are recruited to pre-ribosomes and that recruitment to the pre-90S/40S and pre-60S particles can occur independently.

## DISCUSSION

Has1 was initially identified in affinity purified 90S and early 60S pre-ribosomal particles (9,13,15,18,19) and it was reported that depletion of Has1 led to defects in the synthesis and processing of both 18S and 25S rRNAs (9). In this study, we analyzed the composition of pre-ribosomes purified directly via affinity tagged Has1. In agreement with previously published data, we found that Has1 purifies a mixture of 90S, pre-40S and pre-60S factors. It is most stably associated with the pre-60S particles as evidenced by release of 90S and pre-40S factors during washes with increasing salt concentrations. On the RNA level, wild type Has1 purified 35S, 27SA2, 27SA3, 27SB, 20S and 7S pre-rRNAs, corroborating previous reports (9,16,20). Interestingly, while the experiments in Figure 5 show that binding to the 18S region of the pre-rRNA is weaker compared to binding to 5.8S/25S region, Has1 strongly purifies 20S pre-rRNA. It is possible that Has1 is recruited to the pre-rRNA immediately prior to A2 cleavage, mediates the cleavage and then remains temporarily associated with the freshly generated 20S pre-rRNA. The relatively long life-time of 20S pre-rRNA (37), compared to other intermediates, might also contribute to the apparently strong signal.

The CRAC analysis revealed that wild type Has1 clearly interacts with both 18S/ITS1 and 5.8S/25S regions of pre-rRNAs. The crosslinking pattern of the Has1-DAAD catalytic mutant showed strong reduction in 18S region, in agreement with its inability to bind 90S and pre-40S particles. Our data for the wild type Has1 is in good agreement with the recently published PAR-CRAC analysis (21). Intriguingly, the two distinct sites in the 18S rRNA are rather distant in the 3D structure of the mature 40S ribosome. While the 18S rRNA regions corresponding to these crosslink sites are not currently resolved in the available 90S structures, they are nevertheless not expected to be in close proximity to each other, as the nascent pre-rRNA has been seen rather unfolded apart from the 5′domain in the existing 90S structures (5). It is therefore possible that Has1 is recruited separately to these sites. The observed crosslinking sites in the 5.8S/25S region coincide well with the location of Has1 in several recent cryoEM structures of pre-60S ribosomes (34,38,39) and Figure 3E. In contrast, the resolved parts of the Has1 protein in the available 3D structures (shown also in Figure 3E, F) corresponds to less than a half of the total protein length. Thus, the reach of Has1 is likely to be longer. Furthermore, as an RNA helicase Has1 is probably not static and therefore it is possible that in pre-60S ribosomes the distance of Has1 to all the observed sites is small enough to allow crosslinking. The larger overall binding area of Has1 in pre-60S likely contributes to the stronger affinity of Has1 to pre-60S compared to pre-40S ribosomes observed in the protein purifications. Judging from the multiple contacts in the pre-60s ribosomes, Has1 might assist the folding of the ITS2 region and its processing. However, the catalytic activity is not required for Has1 recruitment to the pre-60S ribosomes as shown both here and by others (20). Additionally, according to Dembowski *et al.* Has1 is also not strictly required for the 27S processing (20). However, one potential caveat to these experiments was that the analysis was performed under conditions where wild type Has1 was depleted, and as such residual wild type Has1 activity cannot be fully excluded. Nevertheless, it is also plausible that the energy of ATP hydrolysis is used for the final release of Has1 from the pre-60S ribosomes, perhaps together with some of the A3 factors.

The binding sites of the wild type Has1 in the 18S/ITS1 region are in close proximity to the binding sites of the pre-40S assembly factors Enp1, Ltv1 and Tsr1 (32). Notably, the same factors co-sedimented with Has1 in approximately stoichiometric ratio in sucrose gradients (Figure 2A). The assembly factors Enp1 and Ltv1 were reported to interact directly and form a complex with Rps3, and are implicated in the late structural reorganization of the pre-40S (40). Interestingly, Tsr1 was implicated in the late steps of processing of 20S pre-rRNA in the cytoplasm (41). However, the localization of Has1 is reported to be nucleolar/nuclear, therefore Has1 must be released from this pre-40S complex before its export to cytoplasm. We do not know whether Has1 mediates other structural changes in this pre-40S complex or participates in the release of the earlier 90S factors after the A2 cleavage. To date, Has1 was not identified in any of the published cryoEM structures of 90S or pre-40S ribosomes, presumably reflecting only transient or flexible nature of its residence in these particles (5,42–44).

Unexpectedly, the catalytically inactive Has1 mutants were able to purify 27SA2 pre-rRNA, which can be produced only in the presence of catalytically active Has1 (Figure 1C). The purification of Has1 catalytic mutants was performed in a strain where both mutant and wild type Has1 were present (without depletion of the wild type Has1 protein). Therefore, there are two possible models that can explain the observed 27SA2 pre-rRNA purification by mutant Has1: (a) the mutant Has1 associates with pre-60S pre-ribosomes containing 27SA2 and replaces the wild type Has1 or; (b) there are two or more molecules of Has1 present at least temporarily in the late 90S pre-ribosomes—one that is required for A2 cleavage (catalytically active) and a second one that mediates 27SA3 processing, which does not require catalytic activity (as shown by (20)). Our data offers multiple lines of evidence in support of the latter model, i.e. the action of two or more independent Has1 molecules during biogenesis: 1. The aforementioned purification of 27SA2 by catalytically inactive Has1 mutants, 2. The presence of Has1 in both pre-60S and pre-40S particles in sucrose gradients and 3. Has1 binding to sites in 18S and 25S rRNAs which are too far apart could be easily explained if different molecules of Has1 were performing these functions. Furthermore, the co-purification of different tagged versions of Has1 strongly implies simultaneous presence of two or more different Has1 molecules in pre-ribosomes. In sucrose gradients, the differently tagged Has1 molecules co-sedimented only with the 90S/heavy pre-60S particles (fractions 9–11 in the Figure 4D). This makes sense as only the 90S pre-ribosomes contain 35S pre-rRNA allowing simultaneous binding of different Has1 molecules to 18S and 5.8S/25S regions. In addition, the quantification of the 3HA and FLAG tagged Has1 molecules in the experiment in the Figure 4A showed that only about a third of 3HA-Has1 present in the input co-purified with the Has1-FTP which served as bait (Figure 4A, B). This fits surprisingly well with the observation that only 30% of pre-rRNA is processed post-transcriptionally in the growing yeast cells and thus 35S pre-rRNA containing 90S pre-ribosomes represent only about a third of all nascent pre-ribosomes (37). All this data supports the model that two (or more) Has1 molecules act independently during ribosome biogenesis.

While we did not detect any Has1-GST in the pre-40S ribosomes in experiments shown in the Figure 4D, the whole experiment involves a long multistep procedure of affinity purification followed by gradient centrifugation, during which weakly associated Has1 molecules might be lost. Therefore, we cannot formally exclude the possibility that multiple copies of Has1 are also present in pre-40S or pre-60S ribosomes. Furthermore, we do not know whether the recruitment of Has1 occurs at different times over the course of biogenesis. The results of the affinity purifications, gradient centrifugation and RNA-protein crosslinking experiments represent only a snapshot of the average occupancy of Has1 at different sites and also survival of the Has1-pre-ribosome complexes through purification procedures. It is also possible that Has1 acts in a ‘touch-and-go’ manner and associates for only a short time with some of its multiple binding sites on pre-rRNA multiple times during biogenesis.

Can Has1 function as a dimer? Has1 was observed to self-interact in the yeast two-hybrid assays (45,46). On the other hand, Has1 identified in the cryoEM structures of pre-60S ribosomes is present as a monomer (34,38,39). It is possible that Has1 is recruited as a dimer but then separates and each monomer functions separately in the pre-40S and pre-60S pathways. We cannot formally exclude this possibility; however, our data do not support the model in which Has1 would be a functional dimer throughout ribosome biogenesis. In the case of a dimer, differently tagged Has1 molecules should co-purify in stoichiometric amounts and also be present in all the fractions of the sucrose gradients (e.g. in Figure 4C), none of which we have observed.

In conclusion, our findings suggest that at least two independent molecules of the Has1 RNA helicase function in ribosome biogenesis and are recruited to separate sites in the 18S rRNA (Has1-18S) and both 5.8S and the 5′end of 25S rRNA (Has1-25S). Has1-18S performs structural rearrangements, in an ATP dependent manner, required for cleavage at the A2 site and remains temporarily associated with the resulting 20S pre-rRNA, following which it is released. The Has1-25S assist in the rearrangements and processing of 27SA2/A3 and is released following the C2 cleavage.

## DATA AVAILABILITY

The mass spectrometry data was deposited to the ProteomeXchange consortium via PRIDE (29) partner repository with the dataset identifier PXD013263.

## Supplementary Material

gkz767_Supplemental_Files
