# Supplementary material for: At least two molecules of the RNA helicase Has1 are simultaneously present in pre-ribosomes during ribosome biogenesis
Source: Nucleic Acids Res. 2019 Sep 12;47(20):10852–64. doi: 10.1093/nar/gkz767 (PMC6846684; doi:10.1093/nar/gkz767)
Supplement: gkz767_Supplemental_Files [file gkz767_supplemental_files.zip › Gnanasundram-SupplementaryFigures_1-6.pdf]

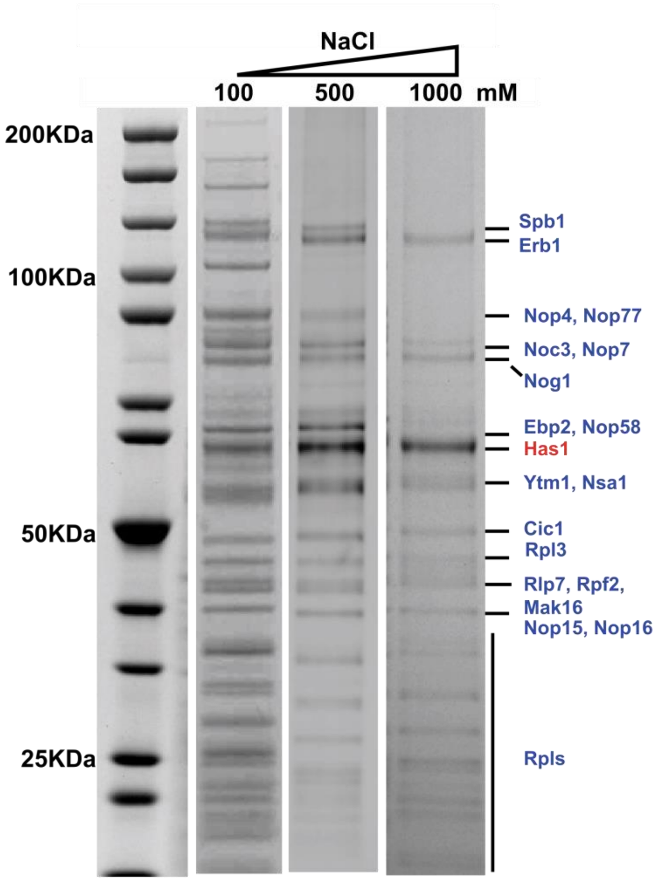

Supplementary Figure 1. Protein composition of the Has1 tandem affinity purification after high salt washing. Has1 affinity purification was carried out with high salt washes before elution with the FLAG peptide. The FLAG eluates were resolved in 4-12% gradient SDS-PAGE and stained with Colloidal Coomassie blue. Proteins retained after high salt washing were identified by mass spectrometry.

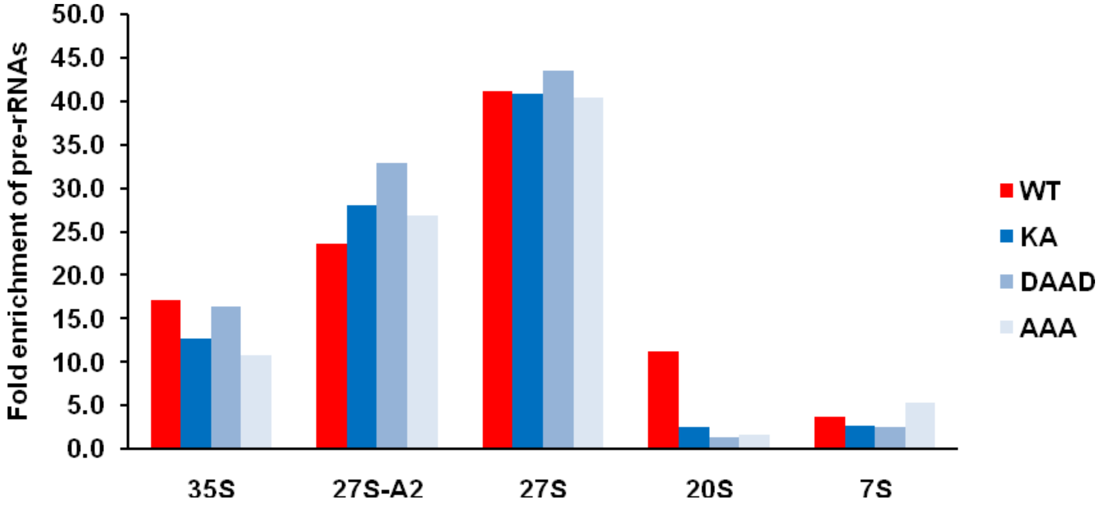

Supplementary Figure 2. A fold-enrichment of pre-rRNAs in the northern blot in the Figure 1C; The enrichment was calculated as a fold over the average signal of 18S and 25S rRNA.

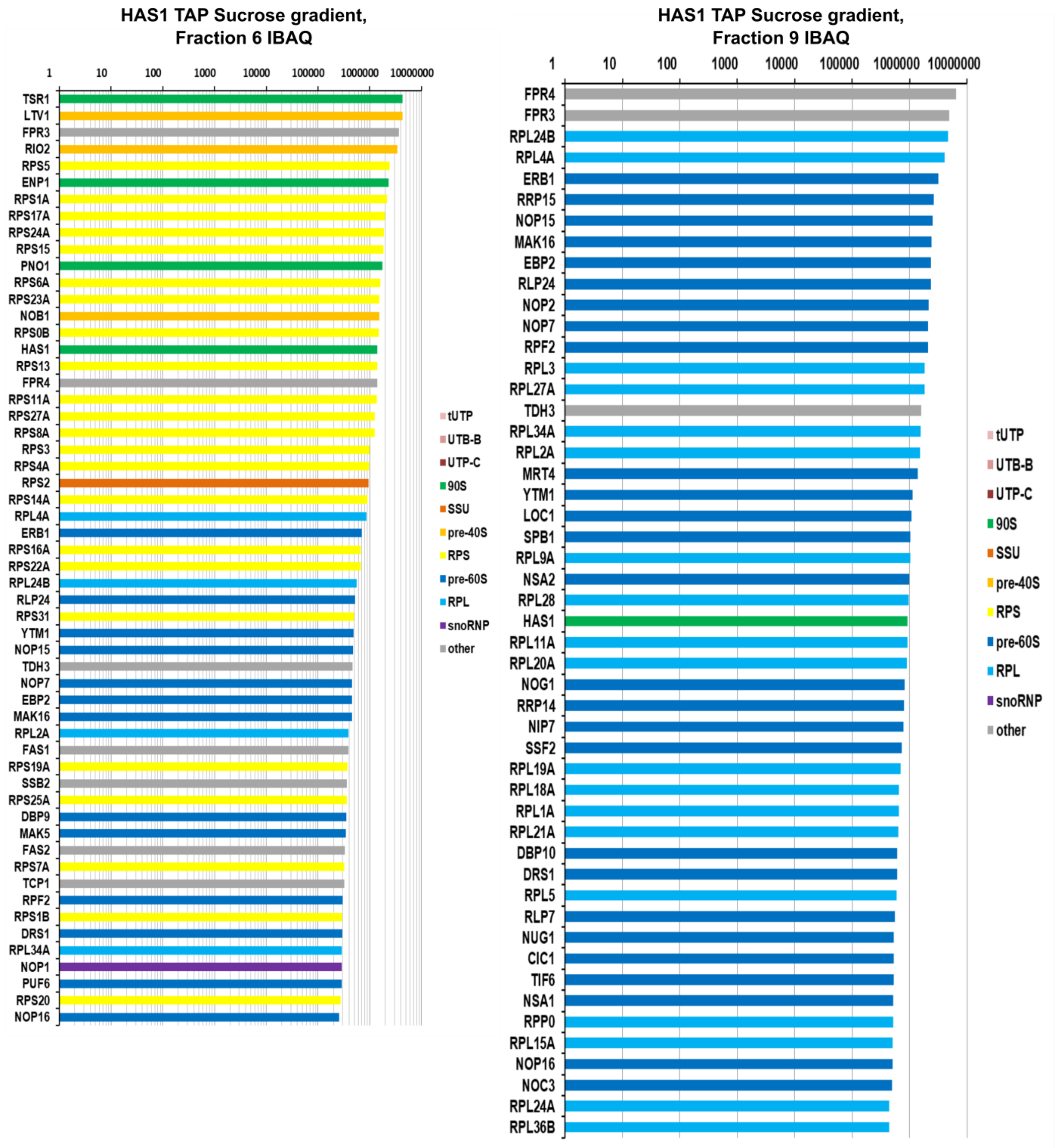

Supplementary Figure 3. Graph showing the iBAQ values of the most abundant proteins in the sucrose gradient fractions 6 and 9. The fraction 6 consisted of primarily pre-40S factors and RPS proteins, while fraction 9 was enriched with pre-60S factors and RPL proteins.

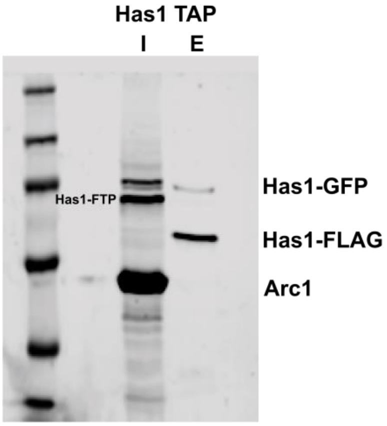

Supplementary Figure 4. Yeast strain expressing Has1-GFP and Has1-FTP was subjected to tandem affinity purification via the FTP tag. The FLAG eluate was then analyzed by Western blotting using the anti-GFP, anti-FLAG and anti-Arc1 antibodies as indicated. Lane I – input, lane E – FLAG eluate.

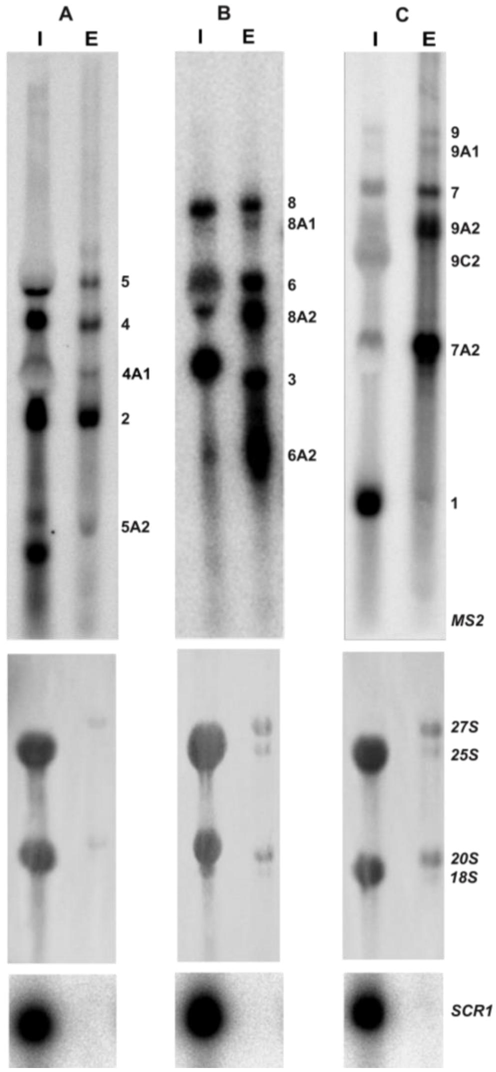

Supplementary Figure 5. Uncropped blots used to produce the main Figure 5B. Has1-FTP was affinity purified from strains expressing different rDNA truncations and the associated RNA was isolated. All the strains were grown to the same OD600 density and a same number of cells was used for each purification. To reduce the number of samples for loading on the northern blot, which would require loading on two gels, we pooled together the RNA purified into three sets of truncations that do not overlap by size. A: pool of samples with DNA truncations 2, 4, and 5; B: pool of 3,6 and 8; and C: 1, 7 and 9. The RNA was pooled only after the full purification and all of the RNA isolated from each truncation strain was used. Lane input (I) was loaded with 1% of input RNA, lane E is the eluate of the RNA purified with Has1.

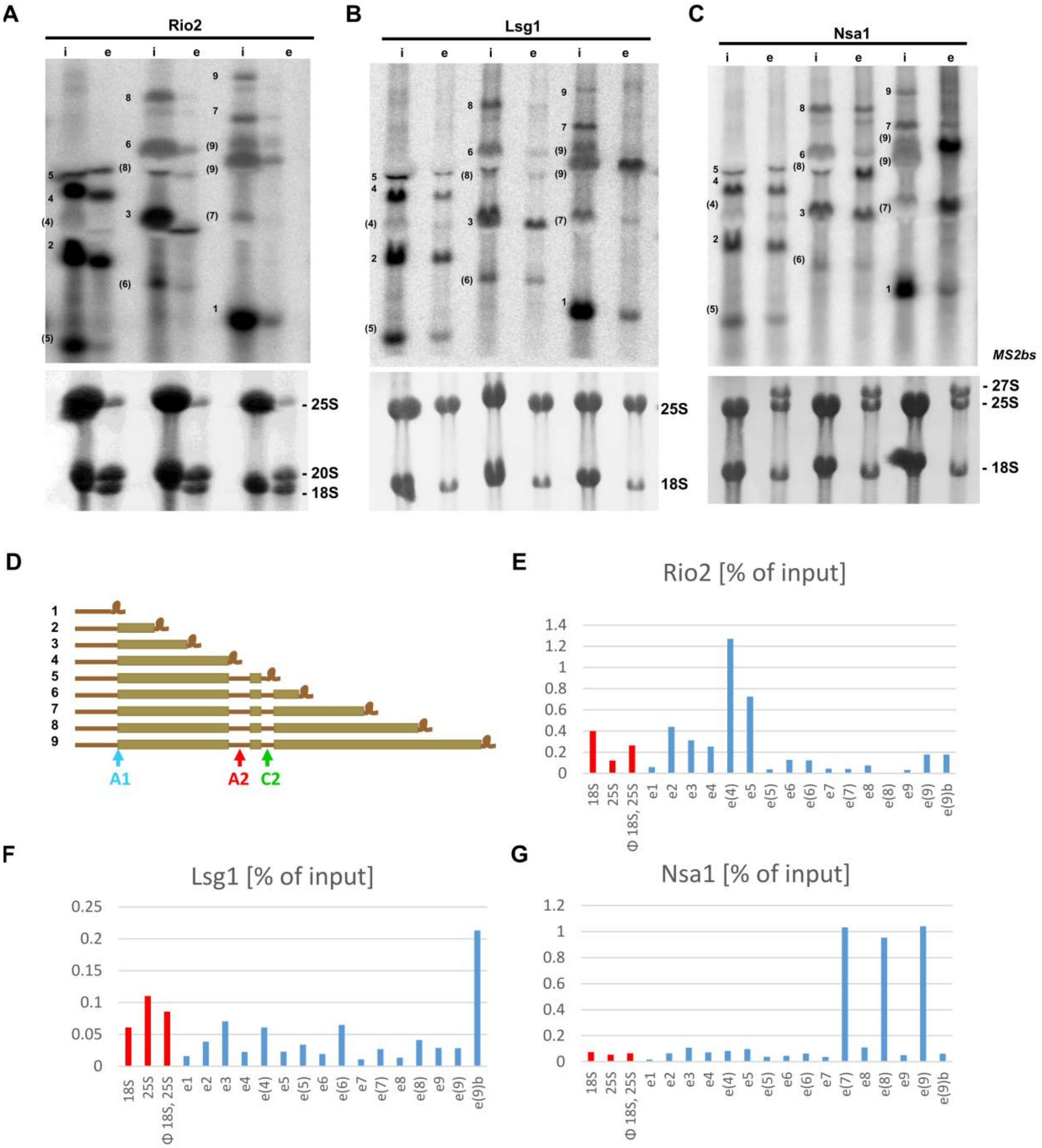

Supplementary Figure 6. Analysis of RNAs associated with different pre-ribosome factors for the estimation of background binding. Northern blot analysis of rRNAs purifying with Rio2 (A), Lsg1 (B) and Nsa1 (C), using the probes against the MS2 tag, 18S and 25S rRNAs. Lane i was loaded with 1% of input RNA, lane e is the total RNA purified with each pre-ribosome factor. D) Schematic representation of the rDNA truncations used. The cleavage sites A1, A2 and C2 are marked on the full-length 35S pre-rRNA construct. E-G) The quantification of the fraction of input purified via Rio2 (E), Lsg1 (F), and Nsa1 (G). Values of the background binding of 18S and 25S rRNAs were in all experiments the highest and therefore used as normalization controls in all the experiments with Has1.
