# Supplementary material for: At least two molecules of the RNA helicase Has1 are simultaneously present in pre-ribosomes during ribosome biogenesis
Source: Nucleic Acids Res. 2019 Sep 12;47(20):10852–64. doi: 10.1093/nar/gkz767 (PMC6846684; doi:10.1093/nar/gkz767)
Supplement: gkz767_Supplemental_Files [file gkz767_supplemental_files.zip › Gnanasundram-SupplementaryTables_3-7.pdf]

Supplementary Table 3: Has1-WT crosslinking sites identified in this study and by Bruning et al., 2018.

rRNA	Crosslinking site	Position in 35S rRNA (nt)	This study		Bruning et al., 2018	Helix	Other factors binding
			**Standard**	**4-TU CRAC**			
**18S**	1	754-801		54-101	71-101	6, 6a	U14 snoRNA
		1107-1128			407-428	13-14	U14 snoRNA
	2	1908-1966	1208-1266			31, 33	ENP1, RIO2
		1922-1977		1222-1277			
		1860-1992			1160-1292	30-35	ENP1, RIO2, NOB1
	3	2012-2058	1312-1358			37-39	LTV1
	4	2119-2211	1419-1511			32, 40, 41	NOB1, LTV1
		2120-2160		1420-1460			
		2114-2167			1414-1467		
**5.8S**	5	2939-2992	78-131			5, 7-9	NOP12
		2925-2997		64-116			
**ITS2**	6	3042-3081		22-61		-	CIC1, NOP15
**25S**	7	3492-3552	241-281			16, 17, 21	ERB1
		3503-3578		252-327			
		3514-3590			263-339		
	8	5791-5806	2540-2555			79	-
	9	6555-6633	3304-3382			98, 101	-

Supplementary Table 4: Has1-DAAD cross linking sites in rRNA

rRNA	Crosslinking site	Position in 35S rRNA(nt)	Has1-DAAD CRAC	Helix	Other factors binding
**18S**	1	1227-1273	527-573	17, 18	-
**25S**	2	3533-3572	282-321	21, 22	ERB1
	3	5397-5470	2146-2219	65, 66	-
	4	5765-5839	2514-2588	79	-
	5	6114-6136	2863-2885	89	-
	6	6555-6608	3304-3357	98, 101	-

Supplementary Table 5: Plasmid constructs used in this study.

Plasmid name	Description	Reference
pRS425	2µ, Episomal, *LEU2*	Christianson *et al.,* 1992
pMK071	pFA6a-GFP(S65T)::natNT2	Van Driessche,B et al., 2005
pMK424	pHTP::HIS3MX6	This study
pMK604	pRS425-*URA3*-*LEU2*d-18S(604nt)-MS2bs-Bo-2 enh-short prom	This study
pMK605	pRS425-*URA3*-*LEU2*d-18S(1142nt)-MS2bs-Bo-2 enh-short prom	This study
pMK606	pRS425-*URA3*-*LEU2*d-18S(1780nt)-MS2bs-Bo-2 enh-short prom	This study
pMK607	pRS425-*URA3*-*LEU2*d-C2-MS2bs-Bo-2 enh-short prom	This study
pMK608	pRS425-*URA3*-*LEU2*d-25S(421nt)-MS2bs-Bo-2 enh-short prom	This study
pMK609	pRS425-*URA3*-*LEU2*d-25S(1453nt)-MS2bs-Bo-2 enh-short prom	This study
pMK610	pRS425-*URA3*-*LEU2*d-25S(2362nt)-MS2bs-Bo-2 enh-short prom	This study
pMK611	pRS425-*URA3*-*LEU2*d-35S-MS2bs-Bo-2 enh-short prom	This study
pMK621	pFA6a-FTP::natNT2	Thoms et al.,2015
pMK623	pRS415-*LEU2*-Has1 WT-FTP	This study
pMK632	pMK140-TetO7-Ubi-Leu-3HA-NatMX4	This study
pMK633	pMK140-TetO7-Ubi-Tyr-3HA-NatMX4	This study
pMK634	pMK140-TetO7-Ubi-Ile-3HA-NatMX4	This study
pMK635	pMK140-TetO7-Ubi-Ala-3HA-NatMX4	This study
pMK774	pFA6a-FTP-HIS3MX4	Kressler et al., 2012
pMK813	pRS425-*URA3*-*LEU2*d-post Dsite-25S(421nt)-MS2bs-B0-2 enh	This study
pMK814	pRS425-*URA3*-*LEU2*d-A3-25S(421nt)-MS2bs-B0-2 enh	This study
pMK815	pRS425-*URA3*-*LEU2*d-5.8S-25S(421nt)-MS2bs-B0-2 enh	This study
pMK816	pRS425-*URA3*-*LEU2*d-E site-25S(421nt)-MS2bs-B0-2 enh	This study
pMK830	pRS425-*URA3*-*LEU2*d-5.8S(155nt)-MS2bs-B0-2 enh-18Stag	This study
pMK831	pRS425-*URA3*-*LEU2*d-C2-MS2bs-B0-2 enh-18Stag	This study
pMK832	pRS425-*URA3*-*LEU2*d-25S (421nt)-MS2bs-B0-2 enh-18Stag	This study
pMK866	pRS415-*LEU2*-Has1 KA-FTP	This study

pMK867	pRS415-*LEU2*-Has1 DAAD-FTP	This study
pMK868	pRS415-*LEU2*-Has1 AAA-FTP	This study
pMK870	pRS425-*URA3*-*LEU2*d-18S-postA2-MS2bs-B0-2enh-18S tag	This study
pMK869	pRS425-*URA3*-*LEU2*d-18S-beforeA2-MS2bs-B0-2enh-18S tag	This study
pMK140	pMK140-TetO7-3HA-NatMX4	This study
pMK014	pFA6a-GST-HIS3MX6	Longtine*et al.,* 1998
pMK711	pRS315-Has1-eGFP	This study
pMK560	pRS415-*LEU2*-FTP	This study

Supplementary Table 6: Yeast strains used in this study.

Strain	Genotype	Plasmid used	Reference
YMK118 (Parental strain)	*MATa; his3Δ1; leu2-3,112; trp1-289; ura3-52; MAL2-8C; SUC2, lys2::tTA, ura3::PCMVtetR'-55, URA3-K.I.*	-	Alexander et al., 2010
YMK444	YMK118, tet::Ubi-Leu-3HA-*HAS1*- NatMX4	pMK632	Gnanasundram et al., 2015
YMK604	YMK118;*HAS1*-FTP:: HIS3MX6	pMK774	This study
YMK539	YMK118; *arg4*Δ, tet::Ubi-Leu-3HA-*HAS1*- NatMX4	-	This study
YMK592	YMK118; *arg4*Δ, tet::Ubi-Leu-3HA-*HAS1*- NatMX4, pRS415-Has1 WT-FTP (*LEU2)*	pMK623	This study
YMK593	YMK118; *arg4*Δ, tet::Ubi-Leu-3HA-*HAS1*- NatMX4, pRS415-Has1 KA-FTP (*LEU2)*	pMK866	This study
YMK594	YMK118; *arg4*Δ, tet::Ubi-Leu-3HA-*HAS1*- NatMX4, pRS415-Has1 DAAD-FTP (*LEU2)*	pMK867	This study
YMK595	YMK118; *arg4*Δ, tet::Ubi-Leu-3HA-*HAS1*- NatMX4, pRS415-Has1 AAA-FTP (*LEU2)*	pMK868	This study
YMK146	YMK118; *ura3Δ::HYG4*	-	This study
YMK688	YMK146; *HAS1*-FTP::natNT2	pMK621	This study
YMK693	YMK146; *HAS1*-FTP::natNT2 , pMK603 (A0- MS2bs-B0-2enh)	pMK603	This study
YMK694	YMK146; *HAS1*-FTP::natNT2 , pMK604 (18S [604nt]- MS2bs-B0-2enh)	pMK604	This study
YMK695	YMK146; *HAS1*-FTP::natNT2 , pMK605 (18S [1142nt]- MS2bs-B0-2enh)	pMK605	This study
YMK696	YMK146; *HAS1*-FTP::natNT2 , pMK606(18S [1780nt]- MS2bs-B0-2enh)	pMK606	This study
YMK697	YMK146; *HAS1*-FTP::natNT2 , pMK607(C2- MS2bs-B0-2enh)	pMK607	This study
YMK698	YMK146; *HAS1*-FTP::natNT2 , pMK608 (25S [421nt]- MS2bs-B0-2enh)	pMK608	This study
YMK699	YMK146; *HAS1*-FTP::natNT2 , pMK609 (25S [1453nt]- MS2bs-B0-2enh)	pMK609	This study
YMK700	YMK146; *HAS1*-FTP::natNT2 , pMK610 (25S [2362nt]- MS2bs-B0-2enh)	pMK610	This study
YMK701	YMK146; *HAS1*-FTP::natNT2, pMK611 (35S-MS2bs-B0-2enh)	pMK611	This study
YMK878	YMK146; *HAS1*-FTP::natNT2 , pMK830 (5.8S[155nt]-MS2bs-Bo-Rnt1-18Stag)	pMK830	This study
YMK879	YMK146; *HAS1*-FTP::natNT2, pMK831(C2-MS2bs-Bo-Rnt1-18Stag)	pMK831	This study
YMK880	YMK146; *HAS1*-FTP::natNT2, pMK832(25S[421nt]-MS2bs-Bo-Rnt1-18Stag)	pMK832	This study

YMK881	YMK146; *HAS1*-FTP::natNT2, pMK813(post D site-25S[421nt]-MS2bs-B0-2enh)	pMK813	This study
YMK882	YMK146; *HAS1*-FTP::natNT2, pMK814(A3 site-25S[421nt]-MS2bs-B0-2enh)	pMK814	This study
YMK883	YMK146; *HAS1*-FTP::natNT2, pMK815(5.8S-25S[421nt]-MS2bs-B0-2enh)	pMK815	This study
YMK884	YMK146;*HAS1*-FTP::natNT2, pMK816(E site-25S[421nt]-MS2bs-B0-2enh)	pMK816	This study
YMK646	YMK118; *HAS1*-HTP::HIS3MX6	pMK424	This study
YMK673	YMK118; pRS415-Has1DAAD-HTP (*LEU2)*		This study
YMK845	YMK118; *HAS1*-eGFP::natNT2	pMK071	This study
YMK261	YMK118; tet::3HA-*HAS1*::natNT2	pMK140	This study
YMK955	YMK118; *HAS1*-GST::HIS3MX6	pMK014	This study
YMK949	YMK146, *HAS1*-FTP::HIS3MX6	-	This study

Supplementary table 7: Oligonucleotides used for Northern hybridization.

Oligo name	Probe used for detecting	Sequence
OMK799	35S	GCTGCTCACCAATGGAATC
OMK800	27SA2-A3	GCAAAGATATGAAAACTCCAC
OMK863	27S A3-B1	GTTCCAGTTACGAAAATTCTTGT
OMK1514	27C1-C2	GTTCGCCTAGACGCTCTCTT
OMK002	20S	CGGTTTTAATTGTCCTA
OMK004	25S	CTCCGCTTATTGATATGC
OMK008	18S	CATGGCTTAATCTTTGAGAC
OMK006	7S	GGCCAGCAATTTCAAGTTA
OMK007	5.8S	GCGTTCTTCATCGATGC
OMK1110	MS2	GTCTTTCTATCGACATGGGTG
OMK215	SCR1	ATCCCGGCCGCCTCCATCAC
OMK1584	18S tag	GAGGATCCAGGCTTTGTC

Supplementary references:

Bruning, L., Hackert, P., Martin, R., Davila Gallesio, J., Aquino, G.R.R., Urlaub, H., Sloan, K.E. and Bohnsack, M.T. (2018) RNA helicases mediate structural transitions and compositional changes in pre-ribosomal complexes. Nat Commun, 9, 5383.

Christianson TW, Sikorski RS, Dante M, Shero JH, Hieter P. (1992). Multifunctional yeast high-copy-number shuttle vectors. Gene 110(1), 119-122.

Van Driessche,B., Tafforeau,L., Hentges,P., Carr,A.M. and Vandenhaute,J. (2005) Additional vectors for PCR-based gene tagging in Saccharomyces cerevisiae and Schizosaccharomycespombe using nourseothricin resistance. Yeast, 22, 1061–8.

Thoms M, Thomson E, Bassler J, Gnädig M, Griesel S, Hurt E (2015). The exosome is recruited to RNA substrates through specific adaptor proteins. Cell 162, 1029–1038.

D Kressler G Bange Y Ogawa G Stjepanovic B Bradatsch D Pratte S Amlacher D Strauß Y Yoneda J Katahira I Sinning E Hurt (2012). Synchronizing nuclear import of ribosomal proteins with ribosome assembly. Science 338, 666-671.

Alexander RD, Barrass JD, Dichtl B, Kos M, Obtulowicz T, Robert MC, Koper M, Karkusiewicz I, Mariconti L, Tollervey D, Dichtl B, Kufel J, Bertrand E, Beggs JD. (2010). RiboSys, a high-resolution, quantitative approach to measure the in vivo kinetics of pre-mRNA splicing and 3'-end processing in Saccharomyces cerevisiae. RNA 16, 2570-2580.

Longtine MS, Fares H, Pringle JR (1998). Role of the yeast Gin4p protein kinase in septin assembly and the relationship between septin assembly and septin function. J Cell Biol 143 (3), 719-736.

Gnanasundram SV, Koš M (2015). Fast protein-depletion system utilizing tetracycline repressible promoter and N-end rule in yeast.MolBiol Cell 26(4), 762-768.
